# Advancements in Sonication-Based Extraction Techniques for Ovarian Follicular Fluid Analysis: Implications for Infertility Diagnostics and Assisted Reproductive Technologies

**DOI:** 10.3390/ijms262110368

**Published:** 2025-10-24

**Authors:** Eugen Dan Chicea, Radu Chicea, Dumitru Alin Teacoe, Liana Maria Chicea, Ioana Andrada Radu, Dan Chicea, Marius Alexandru Moga, Victor Tudor

**Affiliations:** 1 Interdisciplinary Doctoral School, Transilvania University of Brasov, 500019 Brasov, Romania; 2 Clinical County Emergency Hospital Sibiu, 550245 Sibiu, Romania; 3 Faculty of Medicine, Lucian Blaga University of Sibiu, 550169 Sibiu, Romania; 4 Research Center for Complex Physical Systems, Faculty of Sciences, Lucian Blaga University of Sibiu, 550012 Sibiu, Romania; dan.chicea@ulbsibiu.ro; 5 Faculty of Medicine, Transilvania University of Brasov, 500036 Brasov, Romania

**Keywords:** follicular fluid, sonication, proteomics, metabolomics, lipidomics, assisted reproductive technology, biomarker discovery, infertility diagnostics, multi-omics, microfluidics

## Abstract

Ovarian follicular fluid (FF) is a metabolically active and biomarker-rich medium that mirrors the oocyte microenvironment. Its analysis is increasingly recognized in infertility diagnostics and assisted reproductive technologies (ART) for assessing oocyte competence, understanding reproductive disorders, and guiding personalized treatment. However, FF’s high viscosity, complex composition, and biochemical variability challenge reproducibility in sample preparation and molecular profiling. Sonication-based extraction has emerged as an effective approach to address these issues. By exploiting acoustic cavitation, sonication improves protein solubilization, metabolite release, and lipid recovery, while reducing solvent use and processing time. This review synthesizes recent advances in sonication-assisted FF analysis across proteomics, metabolomics, and lipidomics, emphasizing parameter optimization, integration with advanced mass spectrometry workflows, and emerging applications in microfluidics, automation, and point-of-care devices. Clinical implications are discussed in the context of enhanced biomarker discovery pipelines, real-time oocyte selection, and ART outcome prediction. Key challenges, such as preventing biomolecule degradation, standardizing protocols, and achieving inter-laboratory reproducibility, are addressed alongside regulatory considerations. Future directions highlight the potential of combining sonication with multi-omics strategies and AI-driven analytics, paving the way for high-throughput, standardized, and clinically actionable FF analysis to advance precision reproductive medicine.

## 1. Introduction

The analysis of ovarian follicular fluid (FF) has emerged as a key tool in reproductive medicine, offering insight into the biochemical microenvironment surrounding the developing oocyte. FF contains proteins, metabolites, lipids, and signaling molecules that reflect follicular health and potentially predict oocyte quality and In Vitro Fertilization (IVF) outcomes [1,2]. Analytically, FF is a complex and challenging matrix to study. It is essentially a plasma filtrate that is heavily modified by the surrounding granulosa and theca cells, resulting in a unique mixture of components. For example, high-resolution proteomic studies reveal on using techniques such as enhanced filter-aided sample preparation (eFASP) and LCMS/MS, demonstrating its value in biomarker discovery [2].

The success of Assisted Reproductive Technology (ART) critically depends on the ability to select the highest quality oocytes and embryos. While conventional morphological assessment remains the primary method, it frequently lacks the necessary specificity and sensitivity to accurately predict developmental competence, often leading to suboptimal embryo transfer outcomes and cycle cancellation. This limitation has driven a persistent need for non-invasive, molecular biomarkers that can reliably assess oocyte quality in vivo [1]. The FF, as the immediate microenvironment of the oocyte, serves as a direct, dynamic biosensor, reflecting both the systemic status of the patient and the local regulatory processes occurring within the follicle [2]. Consequently, comprehensive molecular profiling of FF offers the potential to move beyond morphological grading towards a personalized, molecular assessment of fertility status and improved clinical prediction of IVF success.

In addition to hormones, FF contains a high concentration of abundant proteins (such as albumin and immunoglobulins), a diverse array of signaling molecules, and a complex lipid milieu, including high levels of lipid–protein complexes and various aggregates [1,3]. This inherent complexity presents significant hurdles for downstream analysis. Specifically, the high concentration of abundant proteins can suppress the signal of lower-abundance biomarkers, while the viscous, heterogeneous nature of the fluid—particularly the presence of lipid aggregates—can interfere with efficient protein solubilization and enzymatic digestion, compromising the overall depth and reproducibility of proteomic, metabolomic, and lipidomic investigations [4,5].

Traditional sample preparation methods for FF proteomics typically involve protein precipitation, centrifugation, and in-solution or on-filter enzymatic digestion. These workflows, though effective, can be cumbersome and time-consuming when analyzing multiple samples [1,3]. Ultrasonication (application of high-frequency acoustic energy to generate cavitation) has been widely used in proteomic workflows to accelerate lysis, enhance protein extraction, and improve enzymatic digestion efficiency in complex biological fluids [4,5]. Its utility has been demonstrated in tissue homogenization, cell lysis, and rapid sample processing for mass spectrometry applications [5].

Sonication-based extraction provides key benefits for follicular fluid analysis, including rapid homogenization, efficient protein solubilization, and improved recovery yields [5]. It also disrupts abundant protein aggregates and lipid–protein complexes within FF [4,6], supporting more consistent extraction outcomes. These advantages are summarized here briefly, with detailed discussion provided in the dedicated section later in the manuscript.

Despite its strong theoretical benefit, the application of sonication to FF analysis is still relatively underexplored. A recent study using human FF in proteomic workflows incorporated ultrasonication in sample rehydration and digestion, improving protein identification and minimizing processing time prior to HPLC/MS analysis (high-performance liquid chromatography–mass spectrometry, which combines chromatographic separation with mass-based detection, enabling precise identification and quantification of complex biomolecules) [1]. However, there remains no standardized protocol for integrating sonication into FF metabolomics or lipidomic analyses, which are increasingly performed using UPLCMS or LCMS methods [7,8].

Emerging research, particularly in metabolomic and lipidomic profiling of FF from women with conditions such as PCOS, diminished ovarian reserve, or ovarian hyperstimulation syndrome (OHSS), underscores the need for robust sample preparation. Metabolomic studies have revealed age-related changes in lipid components like lysophosphatidylcholines and arachidonic acid, which correlate with oocyte competence [8]. Similarly, biomarker profiling in OHSS using UPLCMS identified metabolic shifts that may serve as predictive indicators [6]. Yet, few such studies report using sonication as part of their extraction or processing workflow.

Given the increasing complexity of biomolecular investigations in assisted reproductive research, there is a growing rationale to systematically evaluate sonication-based extraction protocols for FF. These protocols, encompassing protein, metabolite, and lipid analyses, hold the potential to enhance the depth and reproducibility of multi-omic profiling. A carefully validated sonication workflow could not only accelerate the pace of FF investigations but also improve biomarker sensitivity and contribute to the standardization of sample processing across infertility research laboratories.

In this context, the present review provides a comprehensive examination of the field. It first outlines the principles and technical advantages of sonication-based extraction in the analysis of biological fluids. It then examines current applications of sonication in FF research, with particular attention to proteomic, metabolomic, and lipidomic investigations. Recent advances in the identification of FF biomarkers associated with infertility-related conditions, including polycystic ovary syndrome (PCOS), ovarian hyperstimulation syndrome (OHSS), and reproductive aging, are also discussed. Finally, the review addresses the methodological challenges encountered in FF sonication workflows and proposes optimized extraction strategies for FF aliquots within the framework of assisted reproductive technology (ART) research.

## 2. Ovarian Follicular Fluid: Composition and Clinical Relevance

Ovarian follicular fluid (FF) is a metabolically rich and clinically significant biological matrix that mirrors the physiological status of the ovarian follicle and the surrounding oocyte. This chapter explores the biochemical composition of FF, detailing its diverse molecular constituents, including proteins, metabolites, lipids, and signaling molecules, and how these components fluctuate with factors such as age, ovarian pathology, and hormonal environment. Furthermore, we discuss the clinical relevance of FF analysis, emphasizing its role in identifying biomarkers for oocyte quality, infertility diagnostics, and prognostic indicators in assisted reproductive technologies (ART).

### 2.1. Biochemical Composition of FF

Ovarian follicular fluid (FF) is a dynamic and multifaceted medium that surrounds and nourishes the oocyte within the antral follicle. It forms through the passage of plasma components across the theca capillaries, combined with secretions from the granulosa and theca cells [9]. As a result, FF is rich in diverse biomolecules, ranging from plasma derived proteins to metabolites, lipids, hormones, and extracellular vesicles (EVs), that collectively reflect the follicular microenvironment.

Proteomic studies have shown that a significant proportion, nearly 60%, of the proteins present in follicular fluid (FF) are also found in plasma, reflecting the dynamic permeability of the blood–follicle barrier during follicular maturation [9]. Among the most prominent are apolipoproteins (AI, AII, CI, CII, CIII, D, E, F, M), which play central roles in lipid transport and steroid hormone biosynthesis, and complement system components such as C3, C4, and C9, along with coagulation regulators. These proteins are functionally linked to oocyte maturation and fertilization outcomes [9].

Age influences the FF proteome in distinct ways. Comparative analyses between younger and older women reveal marked reductions in proteins such as hemopexin, serotransferrin, and kininogen with advancing age. These declines are associated with reduced antioxidant defense, altered iron homeostasis, and impaired angiogenesis, factors that collectively contribute to diminished oocyte quality and reduced IVF success rates [9].

The metabolomic profile of FF also shifts with age and reproductive pathology. In cases of diminished ovarian reserve, altered concentrations of amino acids and oxidized lipids indicate disrupted energy metabolism and heightened oxidative stress, which correlate with lower oocyte yields and compromised embryo quality [10]. Distinct patterns are observed in conditions such as PCOS and endometriosis, where metabolites including citrate, pyruvate, succinate, aspartate, glutamate, and branched-chain amino acids are significantly altered, reflecting dysregulated carbohydrate and amino acid metabolism [11,12].

Lipidomic analyses further expand this biochemical landscape. In PCOS, FF exhibits elevated concentrations of ceramides and free fatty acids—particularly Cer 36:1;2 and FFA C14:1—along with reduced lysophosphatidylglycerols (LPG 18:0), patterns that are linked to reduced oocyte competence and metabolic imbalance [13]. Age-related lipid changes are also evident; older women show increased levels of LysoPC (16:1), LysoPC (20:4), and LysoPC (20:3), while LysoPC (18:3) and LysoPC (18:1) are reduced, trends consistent with agerelated declines in fertility [12].

Taken together, the biochemical composition of FF provides a detailed reflection of the follicular microenvironment, capturing systemic influences, local follicular development, maternal age, and disease states. This molecular diversity underscores the value of FF as a substrate for comprehensive multiomics profiling aimed at identifying biomarkers of oocyte competence and predicting IVF outcomes.

### 2.2. Clinical Importance of FF Analysis

The analytical examination of ovarian follicular fluid (FF) has emerged as a powerful tool in reproductive medicine, with FF composition providing direct insight into oocyte competence, embryo developmental potential, and ART outcomes. Multiple studies have linked specific FF metabolites, such as glucose, lactate, and pyruvate, with distinct infertility, related conditions, including endometriosis, diminished ovarian reserve (DOR), and polycystic ovary syndrome (PCOS), reflecting altered follicular energy metabolism that correlates with oocyte quality and fertilization success [14,15]. Metabolomic profiling has likewise revealed signature shifts in oxylipins and steroid metabolites in DOR and PCOS patients, which associate strongly with clinical indicators such as AMH, AFC, and fertilization rates [16,17].

From a proteomic perspective, differential expression of proteins such as apolipoprotein D (APOD), paraoxonase 1 (PON1), and heparan sulfate proteoglycans in FF has been shown to predict adverse pregnancy outcomes in subgroups including women with thyroid autoimmunity [18].

Oxidative stress markers measurable in FF—such as malondialdehyde (MDA), total antioxidant capacity, and 8OHdG—also have demonstrated relationships with embryo quality and blastocyst formation, suggesting that an imbalance in reactive oxygen species (ROS) and antioxidant defense may impair ART success [19]. Additionally, FF concentrations of phosphate and other small ions have been statistically associated with ovarian function metrics and live-birth outcomes in IVF studies [20]. Collectively, these findings highlight follicular fluid as a rich, multidimensional diagnostic medium, with its biochemical profiling encompassing integrated proteomic analysis, metabolomics, lipidomics, and redox biology, offering predictive biomarker panels for patient stratification and personalized ART interventions.

## 3. Sample Preparation Challenges in FF Analysis

A rigorous understanding of ovarian follicular fluid (FF) demands high-fidelity analytical methodologies capable of addressing the fluid’s intrinsic complexity, heterogeneous composition, and significant viscosity, all of which vary across patients and clinical conditions. These factors collectively challenge reproducibility, analyte recovery, and downstream sensitivity, especially in proteomic, metabolomic, lipidomic, and extracellular vesicle (EV) analyses. This chapter brings into focus the major barriers encountered in FF sample preparation, beginning with the complexity and heterogeneity of FF samples, before moving on to the limitations of traditional extraction methods and the justification for optimized techniques like sonication-assisted workflows.

### 3.1. Complexity and Heterogeneity of FF Samples

Ovarian follicular fluid exhibits profound biochemical and physical heterogeneity, reflecting contributions from both systemic plasma transudate and paracrine secretion by granulosa and theca cells, alongside dynamic shifts driven by patient age, follicular maturation, ovarian stimulation protocols, and reproductive pathologies such as PCOS and diminished ovarian reserve [21]. This intricate biological matrix contains an abundance of plasma-derived proteins, such as albumin, immunoglobulins, apolipoproteins, and extracellular vesicles (EVs), the latter encompassing a spectrum of particle sizes and cargo profiles. These EVs are frequently isolated by differential ultracentrifugation, though this method often co-isolates contaminating proteins and lipoproteins due to overlapping size and density distributions, thereby complicating downstream proteomic or nucleic acid analyses [22,23].

The colloidal and viscous character of FF adds further operational difficulty. Its non-Newtonian rheology, demonstrated by time-dependent viscosity changes and propensity for spontaneous coagulation-like behavior, interferes with consistent sample pipetting, homogenization, and aliquoting, particularly when volumes are low or lipid content is high [24]. Such viscosity-induced variability introduces technical noise that can mask subtle clinical biomarkers.

Moreover, the dominance of high-abundance proteins and lipoproteins can obscure low-abundance bioactive molecules of clinical interest. These components often form complexes with EVs or metabolites, leading to uneven extraction efficiencies and inconsistent analytical detection across samples. EV preparations can exhibit aggregation artifacts induced by high-speed centrifugation, producing heterogeneous multimodal assemblies that challenge quantification and purity estimation [22,25].

Inter-patient variability further escalates these challenges. Even among normo-ovulatory women, FF composition and viscosity vary significantly, meaning that differences observed across cohorts may reflect both biological variability and technical non-standardization. This underscores the need for robust, reproducible, and high-efficiency sample preparation workflows to ensure that downstream profiling reflects biological reality rather than preanalytical error.

### 3.2. Limitations of Traditional Extraction Methods

Conventional FF sample preparation techniques, such as centrifugation, protein precipitation, ultrafiltration, and ultracentrifugation, often struggle to preserve low-abundance biomarkers and maintain reproducibility. For instance, filter-aided sample preparation (FASP) and enhanced FASP (eFASP), although widely adopted in proteomic workflows, frequently fail to sufficiently deplete high-abundance plasma proteins like albumin and immunoglobulins, thereby limiting the detection of clinically significant low-level proteins in FF [26]. Immunodepletion and fractionation strategies, while potentially improving dynamic range, inherently increase intermediary processing steps and technical variability, especially when applied to limited-volume clinical specimens [27].

Protein precipitation methods, including acetone, trichloroacetic acid (TCA), and chloroform–methanol protocols, can efficiently remove contaminants but often lead to inconsistent recovery of hydrophobic or membrane-associated proteins. These harsh chemical treatments also risk protein denaturation and aggregation, which degrade digestion efficiency and downstream MS detection reliability [28]. For example, post-precipitation pellet solubility issues and low peptide yields are widely reported in workflows involving hydrophobic protein species.

Isolation of extracellular vesicles (EVs) from FF predominantly utilizes ultracentrifugation, considered the “gold standard”; however, EV pellets often co-isolate lipoproteins, protein aggregates, and other contaminants of similar size and density—compromising purity and analytical validity [29,30]. While size-exclusion chromatography (SEC) may yield higher purity in EV preparations, it does so at the expense of recovery and requires additional manual steps that may introduce sample loss and reduce throughput in low-volume clinical settings [30].

Furthermore, critical pre-analytical variables, including centrifugation speed, force, duration, freeze–thaw cycles, and sample handling protocols, are frequently inconsistent across laboratories, contributing substantially to technical variance that can mask true biological signals. These limitations collectively highlight the critical need for streamlined, gentle, and reproducible workflows, such as sonication-assisted extraction, that minimize sample manipulation and optimize yield integrity in FF analysis.

### 3.3. Need for Enhanced Extraction Efficiency and Reproducibility

The limitations of traditional follicular fluid (FF) preparation methods underscore a pressing need for enhanced extraction protocols that maximize analyte yield, minimize technical variability, and preserve molecular integrity across disparate biomolecule types. Sonication-assisted extraction emerges as a compelling solution to these challenges.

Primarily, sonication generates mechanical shear and acoustic cavitation, which can be viewed as shockwaves and microbubbles produced by ultrasonic frequencies, leading to robust disruption of protein–lipid complexes, extracellular vesicle encapsulation, and aggregated colloids. This mechanical process significantly improves the release of low-abundance proteins, metabolites, lipids, and vesicle cargo without resorting to harsh chemical treatments [31]. In comparative workflows involving plant biomatrices, ultrasound-assisted extraction consistently yielded higher proteomic and metabolomic depth, greater recovery of hydrophobic compounds, and faster processing times compared to precipitation- or heat-based protocols, as reported by [32,33].

Moreover, sonication workflows can be standardized and parametrized, with adjustable settings (e.g., frequency, amplitude, pulse duration, duty cycle) that allow reproducible reproducibility across batches and laboratories. Use of calibrated ultrasonic probes or controlled sonication baths with temperature monitoring ensures consistent cavitation efficiency and reduces variability induced by manual processing. Several studies confirm that unlike immunodepletion or precipitation protocols, sonication does not rely on affinity reagents or complex consumables, thereby lowering cost and improving inter-lab harmonization as reported in [34].

Sonication also offers potential for miniaturization and high-throughput automation, making it particularly well-suited for clinical and biobanking settings, where FF sample volumes are often limited. Micro-volume probe sonicators and integrated platforms can process multiple samples in parallel with minimal handling. This capability addresses key limitations of conventional methods, namely, sample loss due to transfer steps and variability across manual protocols.

Moreover, sonication conditions can be tailored to preserve biomolecule stability: using short pulses with cooling intervals prevents overheating and reduces proteome denaturation or oxidative damage. Emerging protocols incorporate temperature control systems and solvent compatibility testing to optimize extraction conditions for metabolomic and lipidomic workflows, ensuring analyte integrity while maximizing yield.

Given these advantages, sonication-based FF preparation workflows hold promise to significantly enhance extraction efficiency, reproducibility, and analytical sensitivity across multi-omic studies, positioning them as a valuable complement or successor to traditional extraction protocols in both research and clinical infertility diagnostics.

**Figure 1:**
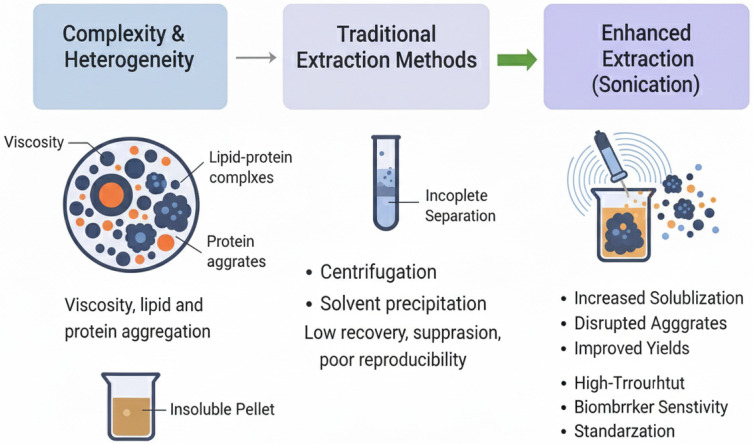
The challenges in sample preparation.

Figure 1 illustrates the challenges in sample preparation.

## 4. Principles of Sonication-Based Extraction

This chapter presents the foundational scientific principles by which sonication enhances extraction efficiency in biological fluids, including its mechanical and physicochemical effects on colloidal and molecular complexity. We first delineate the mechanism of acoustic cavitation, which underlies sonication-based disruption and mass transport enhancement. Subsequent sections will explore optimization parameters, thermal control strategies, and integration within multi-omic workflows tailored to ovarian follicular fluid (FF) analysis.

### 4.1. Mechanism of Acoustic Cavitation

At the heart of sonication lies the phenomenon of acoustic cavitation, in which ultrasonic sound waves (typically 20–100 kHz) propagate through a liquid medium, generating alternating compression and rarefaction cycles. During rarefaction, microscopic cavitation bubbles form in zones of low pressure. As these bubbles grow and subsequently implode during compression cycles, they release intense localized energy characterized by temperatures on the order of 5000 K, pressures approaching 2000 atm, and microjets with velocities up to several hundred meters per second [35,36]. These sono-mechanical effects, shockwaves, shear forces, microstreaming, and turbulence, collectively drive physical disruption of protein–lipid aggregates, extracellular vesicles, and cellular or colloidal structures, thereby enhancing extraction of biomolecules across classes [35,37].

Such cavitation events also induce sono-chemical effects, producing reactive radical species (e.g., hydroxyl radicals), transient hotspots, and accelerated chemical reactions within microenvironments, which can modestly affect certain analytes unless carefully controlled [38]. These combined physical and chemical effects constitute the basis for rapid, high-yield extraction in contexts ranging from botanical compounds to proteins and metabolites.

In the context of FF analysis, acoustic cavitation promotes efficient release of analytes, even from hydrophobic domains or vesicular encasements, while preserving integrity through controlled cavitation parameters. Prosthetically, it enables sono-poration-like effects in extracellular vesicle structures, disrupting membrane barriers without complete degradation, thereby facilitating cargo release (e.g., proteins, nucleic acids, metabolites) [39].

By tuning sonication parameters, frequency, amplitude, pulse duration, and duty cycle, researchers can calibrate the extent of cavitation and shear, balancing extraction efficiency against analyte integrity. High-intensity, low-frequency ultrasound is often preferred for robust sono-mechanical disruption, while higher frequencies can moderate shear effects when delicate components must be preserved.

### 4.2. Sonication Parameters and Optimization

Optimization of sonication parameters is a critical prerequisite for maximizing biomolecule recovery from FF while preserving the biochemical integrity of the sample. Sonication efficiency depends on the interplay of several factors, namely frequency, amplitude (power), pulse duration and duty cycle, total processing time, and thermal control, all of which determine extraction reproducibility and yield [36].

Frequency and amplitude (power) are two important parameters for sonication. The acoustic frequency of the sonicator defines the cavitation regime. Low frequencies (20–30 kHz) promote intense cavitation and mechanical shear, which can efficiently disrupt extracellular vesicles and protein–lipid complexes in FF. In contrast, higher frequencies (>40 kHz) generate gentler cavitation, suitable for workflows aiming to preserve fragile protein conformations [40]. Amplitude, often expressed as a percentage of instrument capacity, controls the energy input into the sample. Standard proteomic sonication protocols, such as those adapted in Clinical Proteomic Tumor Analysis Consortium (CPTAC) workflows, commonly operate at moderate amplitude (40–60%) to balance extraction efficiency with thermal stability [41].

Other important sonication parameters are pulse duration, duty cycle, and cooling. Continuous sonication risks excessive heating, which may denature labile FF components. Pulsed sonication mitigates this by alternating short bursts (10–20 s) with cooling periods (30–60 s) [42]. Optimized proteomic extraction schemes typically apply a total sonication time of 3–6 min, divided into cycles (e.g., six 30 s bursts) interspersed with cooling [41,43].

Sample volume and vessel design are important, as well. Because FF is typically available in small aliquots, low-volume probes (100–500 µL) are preferred. The immersion depth of the probe tip should be carefully maintained to avoid foam formation, which can trap and denature proteins [42]. For high-throughput experiments, multi-sample sonicators such as the UIP400MTP enable parallel processing of FF aliquots under controlled reproducible energy conditions [35].

Not neglecting at all are buffer composition and protein solubility. The buffer system supports both mechanical disruption and protein stability during sonication. Urea (8 M) and mild detergents (e.g., 0.1% SDS) are widely applied to maintain solubility and prevent protein aggregation during cavitation [42,44]. Protein-rich samples may require longer or repeated sonication cycles, although excessive energy input risks peptide fragmentation [44].

Optimization of the workflow plays a crucial role in the quality of the sonication process. An iterative approach is recommended for parameter optimization: equal FF aliquots are processed under varying pulse lengths (30, 60, 90 s) at fixed amplitude while monitoring sample temperature. Extraction yield is assessed via marker proteins (e.g., ACPP, CD5L) and integrity validated through downstream proteomic assays.

Another important parameter is the thermal control. Active thermal management, typically using an ice-water bath or a temperature-controlled sonication platform, is essential to prevent overheating. A practical benchmark is to limit total sonication energy to 4–6 kJ/mL to minimize denaturation, especially in FF samples rich in low-molecular-weight peptides and lipoprotein complexes, as stated by [40,42].

The optimized ranges for these parameters are summarized in Table 1.

A question naturally rises concerning the sonication, related to sonication vs. detergent lysis. Evidence from proteomic workflows suggests that ultrasonication alone can achieve comparable, and in some cases superior, release of membrane-associated proteins compared to detergent-only lysis, provided that thermal control is maintained and cavitation intensity is optimized [35,44,45].

### 4.3. Advantages of Sonication over Conventional Methods

Sonication-assisted extraction offers remarkable advantages over traditional FF preparation techniques, especially centrifugation, solvent precipitation, and ultrafiltration, by enabling rapid homogenization, enhanced recovery of biomolecules, and minimal solvent consumption.

Firstly, rapid homogenization is achieved via acoustic cavitation: collapsing microbubbles generate intense localized shear forces that disrupt complex matrices and accelerate mass transfer into the solvent phase. This dramatically reduces extraction time compared to extended centrifugation or precipitation protocols that rely on passive diffusion [45].

Secondly, sonication significantly enhances protein and metabolite release. Studies on plant and algal systems have reported protein recoveries equivalent to overnight maceration—in minutes rather than hours—and even yield increases of ~1.3 fold over conventional methods [46,47]. Though these are not from FF per se, the underlying mechanism, intense local disruption followed by solvent penetration, translates to complex biological fluids like FF. This improved extraction efficiency also applies to low-molecular-weight metabolites and lipids, with higher sensitivity for downstream LCMS workflows.

Finally, sonication is inherently a green extraction technique, requiring minimal solvent volume. Its efficacy with lower solvent-to-sample ratios reduces hazardous waste and cost, which is consistent with broader trends in green chemistry and sustainability in analytical workflows [46].

These combined advantages make sonication-based extraction particularly appealing in the context of FF analysis, where sample volumes are limited, reproducibility is critical, and preservation of biomolecule integrity is essential. Table 2 summarizes the advantages of sonication-based extraction

### 4.4. Potential Artifacts and Mitigation Strategies

While sonication-based extraction offers clear analytical benefits, it may introduce artifacts, notably sample heating, protein denaturation or aggregation, and reproducibility challenges, that must be actively addressed.

Sample heating is one main concern in sonication. Sonication generates local heat via acoustic cavitation, especially under prolonged or high-power settings. Elevated temperatures (>30–35 °C) can accelerate protein unfolding and degradation, undermining biomolecule integrity [35]. Temperature spikes are particularly problematic in sensitive matrices like FF, where native structures and enzymatic states are clinically informative. To control thermal buildup during sonication, the process should be performed in pulse mode, using short bursts (e.g., 5 min with cooling intervals), while maintaining the samples in an ice bath or temperature-controlled holder to ensure the bulk temperature remains below critical thresholds (≤30 °C) [35].

Another concern is related to protein denaturation and aggregation. Mechanical shear and sono-chemical effects may induce conformational changes in proteins. Studies have documented amyloid-like aggregate formation, with increased βsheet content and resistance to resolubilization, even for diverse proteins like BSA or lysozyme [48]. Excessive amplitude or duration further promotes unfolding and loss of native function [49]. Mitigation of this is done by optimizing frequency, amplitude, and duration to balance yield vs. structural preservation. Proteins with significant α-helical content are especially vulnerable; mild settings reduce aggregation risk [48]. Monitoring via circular dichroism or SDSPAGE can guide parameter selection.

Reproducibility in sonication-based extraction remains a critical challenge, as even small deviations in sonotrode immersion depth, sample volume, thermal regulation, or cycle timing can introduce variability between runs. Uncontrolled heating and inconsistent cavitation fields further exacerbate inter-sample differences, sometimes occurring even within the same laboratory environment [50]. These limitations can be effectively managed through standardized operating protocols that fix sonotrode positioning, amplitude, and cycle parameters, coupled with continuous temperature monitoring, preferably using sensors or thermocouples with real-time feedback control, to prevent thermal drift [49]. When feasible, inclusion of technical replicates and instrument blanks can help detect systematic biases.

By embedding these controls into the workflow, sonication can be applied to FF with consistency and precision, ensuring its suitability for high fidelity downstream analyses in ART biomarker research. Table 3 summarizes the artifacts and mitigation strategies for sonication in FF extraction.

**Figure 2:**
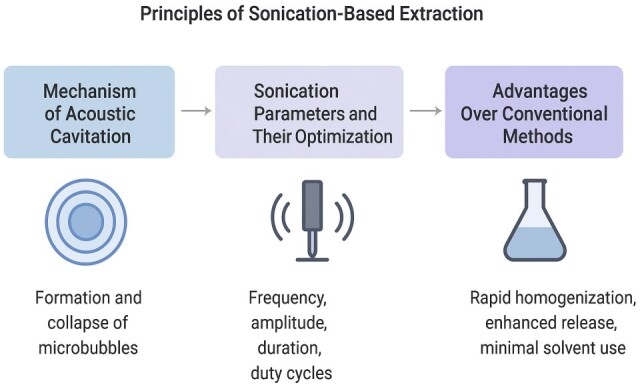
The principles of sonication extraction.

The principles of sonication extraction are summarized in Figure 2 below.

## 5. Applications of Sonication in FF Proteomics, Metabolomics, and Lipidomics

As the field of infertility diagnostics pushes toward precision medicine, the comprehensive multi-omics analysis of ovarian follicular fluid (FF) has emerged as a critical frontier. Sonication-assisted extraction is becoming a cornerstone in preparing FF for proteomic, metabolomic, and lipidomic workflows by efficiently disrupting this complex biological matrix to release a rich repertoire of biomolecules. In this chapter, we explore how sonication enhances each omics layer, starting with proteomics, where improved solubilization and digestion efficiency can significantly expand the detectable proteome and deepen insight into oocyte microenvironments relevant to assisted reproductive technologies.

### 5.1. SonicationEnhanced Proteomic Analysis of FF

Sonication greatly improves protein solubilization and digestion efficiency in FF proteomics workflows. Conventional methods often rely on solvent precipitation, centrifugation, or extensive incubation to denature and solubilize proteins, steps that can limit detection of low-abundance species. In contrast, sonication directly disrupts FF’s complex lipid–protein and extracellular vesicle matrices, facilitating rapid solubilization in lysis buffers and promoting more homogeneous dispersion [1]. Thawed FF proteins precipitated and subsequently resuspended via ultrasonication (20% power, 50% pulse, on ice) achieved consistent protein concentrations (~150 µg) prior to enzymatic digestion, underscoring both efficiency and reproducibility [1]. Moreover, improved release of proteins enhances downstream enzymatic digestion, reducing missed cleavage, increasing peptide yield, and improving identification sensitivity in LC–MS or UHPLC–MS/MS pipelines. Prior FF proteomic studies using SDS-PAGE, OFFGEL and SCX-based separation followed by LC-MS/MS analysis to characterize the proteome of human follicular fluid. The study [51] reported high confidence identification of 480 proteins, of which 320 have not been described previously to the date of publication in the follicular fluid and tagging workflows. The incorporation of sonication can further expand both depth and consistency of proteome coverage, including low-abundance signaling molecules and potential biomarkers of oocyte competence or ovarian disorders. Table 4 illustrates the benefits of sonication in FF proteomic workflows.

### 5.2. SonicationAssisted Metabolomic and Lipidomic Profiling of FF

The integration of sonication into metabolomic and lipidomic extractions significantly enhances the recovery and breadth of small molecules from follicular fluid. By disrupting extracellular vesicles and lipid-rich microstructures, sonication improves release and solubilization of both polar and nonpolar metabolites and lipids into extraction solvents. This leads to greater sensitivity and reproducibility in downstream LC–MS or UHPLC–MS/MS analyses, vital for capturing subtle biochemical differences linked to infertility or ovarian disorders.

Clinically, untargeted metabolomic studies in FF have identified key amino acids (e.g., proline, glycine, arginine, threonine), glucocorticoids, and energy intermediates as predictors of oocyte competence or endometriosis status [52,53]. For example, in normal-weight PCOS patients, LCMS profiling revealed dysregulation in glycerophospholipid and steroid metabolism, including elevated prostaglandin E_2_ and lysophosphatidylcholines (LPCs), with strong predictive value (AUC ≈ 0.80) [54]. Lipidomic surveys in OHSS and PCOS contexts uncovered altered levels of cholesterol esters, phosphatidylinositols, sphingomyelins, and triglycerides linked to poor IVF outcomes [7,54]. Similarly, age-related FF lipid shifts (e.g., increased arachidonate, lysoPC variants) correlate with diminished oocyte quality in older women [54]. Sonication aids detection of these low-abundance metabolites by improving extraction efficiency.

Standard protocols involve mixing FF with cold methanol or acetonitrile, then applying brief sonication (e.g., in an ice bath, 510 min). Following centrifugation or precipitation, supernatants are analyzed via LC–MS or UHPLC–MS/MS. This enhances detection of hydrophilic amino acids, steroids, phospholipids, sphingolipids, and fatty acids, increasing metabolite coverage and reducing signal variability.

These combined metabolomic and lipidomic insights are essential for uncovering biomarkers related to oocyte quality, PCOS, endometriosis, OHSS, and age-related ovarian decline. Sonication-based extraction enhances sensitivity, depth, and reproducibility, bridging sample-limited FF workflows with high-impact infertility diagnostic research. Table 5 presents the sonication benefits in FF metabolomics & lipidomics.

### 5.3. Lipidomic Applications of Sonication in FF Extraction

Lipidomics of follicular fluid (FF) holds significant promise for identifying biomarkers linked to ovarian pathology and IVF outcomes. Sonication enhances traditional lipid extraction protocols (e.g., Bligh & Dyer, Folch), especially when dealing with limited FF volumes and diverse lipid classes. The disruption of extracellular vesicles and lipid–protein complexes via sonication improves lipid solubilization into organic phases, improving both yield and reproducibility in sensitive LC–MS or UHPLC–MS/MS workflows.

In controlled studies, lipidomic profiling using ESI-MS or UHPLC-HRMS has revealed differential abundance of phospholipids (phosphatidylcholines, phosphatidylinositols), sphingolipids, triglycerides, cholesterol esters, and lysophospholipids in FF from women with endometriosis, PCOS, OHSS, unexplained infertility, and age-related decline [55,56]. For example, in endometriosis cohorts, elevated sphingomyelin and phosphatidylcholine species distinguished affected from control groups [55]. In PCOS patients, altered glycerophospholipid metabolism, particularly elevated prostaglandin E_2_ and LPC levels, achieved predictive AUC ≈ 0.80 for oocyte competence [54]. Age-related lipid shifts include increased arachidonic acid and Lyso-PC species in older women, correlating with diminished oocyte quality [56].

While many published FF lipidomic studies employed Bligh & Dyer [57] without sonication like [54,55], incorporation of sonication prior to phase extraction is now recommended to enhance lipid recovery, especially for low-abundance and labile species. Standard workflows involve mixing FF with chloroform/methanol/water, followed by brief ice bath sonication (3–5 min) to disrupt vesicles and protein complexes, then centrifugation and phase separation for MS analysis. This improves both lipid yield and consistency across sample batches.

Given the clinical relevance of FF lipid profiles in infertility phenotypes, sonication coupled with modern lipidomics provides a scalable and sensitive approach to uncovering lipid biomarker candidates in ART and reproductive medicine. Table 6 presents in a concise manner the sonication benefits in FF lipidomic profiling.

### 5.4. Integration with Mass Spectrometry Workflows

Integration of sonication-enhanced extraction with advanced mass spectrometry platforms has transformed follicular fluid (FF) analysis, delivering deeper proteomic, metabolomic, and lipidomic insights. Sonication streamlines sample preparation, enabling more complete protein solubilization, efficient metabolite and lipid release, and uniform extraction across small-volume samples, thereby maximizing the sensitivity and reproducibility of both LC–MS and UHPLC–MS/MS analyses.

In bottom-up proteomics, ultrasonication markedly improves protein extraction and digestion efficiency. Studies utilizing liquid chromatography–tandem mass spectrometry (LC–MS/MS) for FF analysis identified 1392 proteins when using optimized eFASP protocols with SDS denaturation, highlighting FF’s proteomic complexity and the need for robust sample preparation [58]. Sonication further enhances such workflows by improving protein yield and digestion completeness, essential for high-depth proteome mapping.

For metabolomics and lipidomics, sonication is applied early in extraction, typically through ice-bath sonication of FF with cold methanol/acetonitrile or MTBE solvent mixes. This disrupts extracellular vesicles and lipid–protein aggregates, releasing polar and nonpolar analytes into solution. In PCOS FF studies, MTBE extraction combined with sonication (20 min at 4 °C) followed by UHPLC–MS/MS enabled high-resolution profiling of over 800 lipid species and clear discrimination of aberrant lipid signatures [59,60]. Notably, UHPLCESIHRMS analysis of OHSS cohort samples revealed characteristic shifts in lysophosphatidylcholine, phosphatidylinositol, sphingomyelin, triglycerides, and cholesterol esters—lipids whose efficient extraction is aided by sonication [60].

Reversed-phase UHPLC–MS, widely used for profiling biological fluids and tissues in studies of lipid dysregulation, is limited in conventional systems by fluidic dispersion and thermal gradients. In a recent study [61], vacuum jacketed columns placed at the MS source improved lipidomic analysis of plasma extracts by delivering 66% higher peak capacity, up to 34% less peak tailing, 30% more lipid detections, doubled peak intensity, and 22% more identifications, while maintaining reproducibility (1.8–12% RSD) and stable retention times in pooled QC samples.

On the high-throughput front, modern mass spectrometry platforms support cohort-scale studies of FF. Data-independent acquisition (DIA, which is a mass spectrometry method that fragments all ions across defined mass ranges, enabling unbiased, comprehensive, and reproducible analyte detection) and tandem mass tag (TMT, which is a chemical labeling technique that enables simultaneous identification and precise quantification of proteins from multiple samples in a single mass spectrometry run) workflows can quantify thousands of proteins per batch with high reproducibility (CV < 5%)—however, uniform extraction across samples is crucial. Automated sonication systems integrated into liquid handling platforms ensure consistent protein and metabolite release across large sample sets, minimizing inter-well variability and supporting robust biomarker discovery. While few FF-focused studies explicitly cite automated sonication, analogous high-throughput proteomics frameworks have shown that standardized sonication across plates dramatically reduces technical noise [62].

Similarly, shotgun lipidomics, direct infusion ESI of crude extracts, facilitates rapid profiling of hundreds of lipid species from minimal volumes, and relies on sonication-based disruption for clean, reproducible extraction. Combined with UHPLC–MS/MS runs of less than 15 min, these workflows enable high-sample throughput essential for clinical cohorts.

Together, these integrations illustrate how sonication not only enhances extraction efficiency but also underpins the reliability and depth of mass spectrometry-based analyses in FF omics studies. The synergy enables expansive biomolecule coverage, cleaner spectral data, and scalable workflows vital for infertility diagnostics and assisted reproductive technology research. Table 7 illustrates in a concise manner the benefits of sonication coupled with mass spectrometry for FF analysis

**Figure 3:**
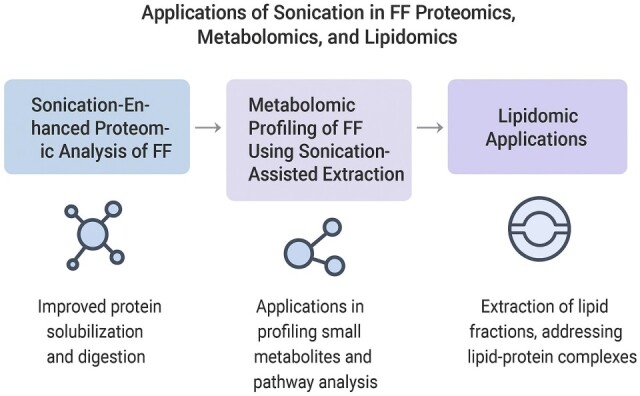
Applications of sonication in FF proteomics, metabolomics, and lipidomics.

Figure 3 below illustrates the applications of sonication in FF proteomics, metabolomics, and lipidomics.

## 6. Emerging Techniques: Sonication Combined with Microfluidics and Automation

As fertility diagnostics advance toward miniaturized, high-throughput and point-of-care platforms, emerging technologies that combine sonication with microfluidics and automation are poised to revolutionize FF analysis. This chapter explores three converging trends: integration of sonication into microfluidic formats to process minute FF volumes; incorporation of automated high-throughput sonication systems for batch processing; and the potential deployment of compact, onsite or point-of-care (POC) devices for rapid, clinic-ready biomarker extraction and analysis.

### 6.1. Microfluidic Sonication Devices for SmallVolume FF Analysis

Microfluidic platforms excel at handling nanoliter to microliter sample volumes—an essential feature when working with precious follicular fluid (FF) derived during IVF procedures. Incorporating sonication into microfluidic channels further enhances this capability by efficiently disrupting extracellular vesicles, protein aggregates, and lipid-rich domains within ultralow FF volumes, facilitating improved biomolecule release into extraction solvents.

An application of this principle is in circulating cell-free DNA extraction from sub-milliliter plasma using acoustofluidic microfluidics. Here, bubble-based micromixers driven by ultrasound significantly improved extraction efficiency, up to tenfold higher than control methods, demonstrating how localized acoustic energy can enhance recovery even in tiny volumes [63].

Another example lies in creating liposomal nanoparticles via microfluidic devices equipped with sonication modules. By incorporating ultrasound within the microchannel, ref. [64] reports on achieving finer and more uniform liposome distributions, highlighting how ultrasound in microfluidic flow can control particle size with precision.

Moreover, epigenetic assays on microfluidic chips integrating sonication demonstrate that acoustic streaming in tiny chambers not only facilitates efficient shearing of chromatin or DNA but also enhances reagent mixing and washing steps. This integration enables sensitive, rapid chromatin immunoprecipitation (ChIP) and methylated DNA immunoprecipitation (MeDIP) from starting materials of only a few thousand cells, with entire workflows completed in under an hour within microscale environments [65].

These studies, while not FF-specific, lay the foundation for adopting sonication-equipped microfluidic devices for FF sample preparation. Forward-designing such systems could enable rapid, low-volume extraction of FF proteins, metabolites, nucleic acids, and lipids with remarkable efficiency and minimal material loss, critical in clinical ART and infertility biomarker research. Table 8 summarizes the key developments in microfluidic sonication platforms.

### 6.2. HighThroughput Automated Sonication Systems

In modern omics research, particularly in reproductive medicine, processing dozens or hundreds of FF samples demands both speed and precision. High-throughput automated sonication systems fulfill this need by combining focused acoustic energy with plate-based workflows and robotic control. Notably, systems like Hielscher’s UIP400MTP (Teltow, Germany) and Covaris’s AFA^®^ high-throughput focused-ultrasonicators (Woburn, MA, USA) facilitate reproducible disruption of hundreds of FF aliquots simultaneously, all while supporting standard laboratory automation.

The UIP400MTP applies focused ultrasonication to multi-well plates, ranging from 12- to 1536-wells, via non-contact cup-horn sonotrode design. Its uniform acoustic field ensures consistent cavitation and homogenization across all wells, as validated by aluminum foil and emulsion tests that confirm homogeneous sonication intensity and reproducibility [66]. Temperature sensors and programmable control over amplitude and pulse cycles prevent sample overheating, preserving the integrity of biomolecules in FF extracts. Additionally, the UIP400MTP integrates seamlessly into automated workflows and standard autosampler lines, eliminating laborious manual interventions and mitigating cross-contamination risks by accepting any standard plate format [66].

On the AFA^®^ front, Covaris’s LE220-plus, LE220R-plus, and R230 on-deck focused-ultrasonicators (Woburn, MA, USA) exemplify high-throughput sonication tailored for integration with robotic automation. These systems accommodate 1–96 samples per batch and offer precise control over acoustic energy, enabling high reproducibility essential for multiplexed FF analysis [67]. The robotic-friendly R230 enables direct placement on liquid-handling decks, allowing automated, walk-away FF preparation workflows, a significant advantage in clinical reproductive laboratories.

Such automated platforms have been embraced in other fields, for example, Covaris’s Sonication STAR (Woburn, MA, USA) has been successfully integrated with Hamilton liquid handlers (Reno, NV, USA) to automate deparaffinization and nucleic acid extraction from FFPE tissues, boosting sample throughput and reducing failures due to insufficient material [68]. Translating this level of automation to FF workflows could equally streamline proteomic, metabolomic, and lipidomic sample preparation, enhancing both speed and consistency in infertility biomarker studies. Table 9 summarizes the high throughput automated sonication for FF analysis.

### 6.3. Potential for PointofCare or OnSite Applications

As infertility care moves toward personalized, rapid-response strategies, the development of point-of-care (POC) or on-site platforms for follicular fluid (FF) analysis promises transformative clinical impact. These systems aim to deliver “samplein, answerout” capabilities directly within IVF clinics or procedure rooms, reducing dependency on centralized labs, accelerating decision-making, and conserving precious FF volumes. Leveraging microfluidic integration, POC devices can incorporate sonication steps, streamline multi-omic extraction, and interface with mobile detection modules, all within a compact, user-friendly tool. This subsection highlights the key technologies enabling such innovations and discusses how they can be adapted to FF workflows.

Microfluidic POC frameworks provide the foundational architecture for integrated, miniaturized diagnostic devices. Their ability to perform sample manipulation, analyte separation, and detection in a single enclosed environment makes them especially suitable for sonication-enhanced FF workflows. Microfluidic POC systems are characterized by low reagent consumption, rapid turnaround, and portability, all critical for incorporating FF biomarker analysis within clinical settings [69,70].

“Labonachip” devices have demonstrated the potential to process complex biofluids by integrating sample handling, molecular reaction stages, and readout into a sterilizable micro-platform, achieving results within minutes [70]. Although not yet applied directly to FF, similar systems could incorporate localized sonication zones to enable efficient homogeneous release of proteins, metabolites, lipids, or extracellular vesicles from micro-volume FF, all within a closed cartridge format.

Moreover, such platforms can be paired with smartphone-based detection systems. Wearable or handheld microfluidic devices now interface with smartphone cameras or sensors for optical readout, enabling real-time data capture and remote results communication [69]. In an IVF clinic, this could translate to same-session insight into oocyte competence biomarkers, enabling immediate clinical decisions. Collectively, POC microfluidic devices equipped with sonication capabilities offer compelling prospects: ultra-low sample volume processing, integrated extraction-to-detection workflows, rapid analytics, and scalable translation to clinical ART practice. Table 10 summarizes the POC microfluidic devices with sonication for FF analysis.

## 7. Clinical Implications and Diagnostic Potential

This chapter examines the translational significance of integrating sonication-based extraction into follicular fluid FF analysis, detailing how methodological advancements catalyze deeper biomarker discovery, enable personalized infertility interventions through real-time FF analysis, and bridge the gap between bench research and clinical ART workflows. The emphasis lies on translating analytical innovation into improved diagnostic precision, therapeutic decision-making, and ultimately, patient outcomes in infertility treatment.

### 7.1. Improving Biomarker Discovery Pipelines

Optimizing biomarker discovery in follicular fluid (FF) hinges on both the detection sensitivity for low-abundance analytes and reproducibility across samples. Sonication-based extraction plays an important role by ensuring efficient disaggregation of extracellular vesicles, lipid–protein complexes, and dense macromolecular assemblies within FF. These improvements enhance the release of discrete biomolecules, encompassing metabolites, lipids, and proteins, thereby amplifying their visibility in downstream mass spectrometry workflows.

Recent metabolomic analyses highlight the transformational impact of improved sensitivity and consistency. For example, in a comparative profiling study, infertile women undergoing IVF exhibited distinct lipid perturbations in their FF, including elevated levels of phosphatidic acid, phosphatidylinositol, monogalactosyldiacylglycerol, phosphatidylglycerol, sphingomyelin, diacylglycerol, and triacylglycerol, relative to fertile controls. These differential lipid signals were robustly detected using SWATH-to-MRM metabolomics with excellent quantitative precision [7]. In parallel, targeted investigations identified lysophosphatidylcholine (LPC) as a highly predictive marker of follicular developmental competence. Notably, LPC levels correlated strongly with oocyte quality, and findings were validated via enzyme assays across large sample sets, underscoring considerable reproducibility in detection [71].

Beyond lipids, metabolomic surveys in diverse infertility contexts, such as endometriosis or diminished ovarian reserve, have consistently revealed alterations in glucose, lactate, pyruvate, and choline metabolite patterns in FF [72], reinforcing both the sensitivity and the relevance of these profiles in clinical phenotyping. Improved extraction reproducibility is pivotal here, consistent release of metabolites underlies dependable, statistically meaningful group comparisons.

In summary, sonication-enhanced FF workflows significantly elevate both sensitivities, capturing minute yet informative molecular signatures, and reproducibility, ensuring data consistency across cohorts. Together, these gains strengthen the foundation for discovering actionable infertility biomarkers capable of robust validation and eventual clinical translation. Table 11 summarizes the sonication-driven enhancements in biomarker discovery.

### 7.2. Role in Personalized Infertility Treatment

The evolution of personalized medicine has begun to permeate the field of reproductive health, particularly in assisted reproductive technologies (ART). One of the emerging frontiers in this domain is the use of real-time follicular fluid (FF) analysis to guide individualized treatment protocols during in vitro fertilization (IVF). With the advent of advanced analytical technologies and minimally invasive procedures, it is now conceivable to integrate rapid FF biomarker assessment into clinical workflows, offering actionable insights at the time of oocyte retrieval.

Several studies have demonstrated the potential of FF biomarkers to predict oocyte quality and developmental competence. For instance, leptin, a hormone involved in energy regulation, has been shown to correlate with oocyte maturity. A recent study reported that FF leptin levels were significantly higher in women with mature oocytes, with a proposed threshold of 16 ng/mL achieving 70% sensitivity and 91% specificity (AUC = 0.829) for predicting maturation [73]. This underscores leptin’s potential as a point-of-care biomarker for evaluating oocyte competence before fertilization.

Lipid mediators, particularly Resolvin E1 (RvE1), have also emerged as promising indicators of oocyte quality. A targeted metabolomic analysis revealed that lower FF concentrations of RvE1 were associated with poor-quality oocytes, and levels below 8.96 pg/mL provided 97% specificity (AUC = 0.75) for predicting suboptimal oocytes [74]. Given that sonication enables the rapid release of lipid species without compromising structural integrity, this method may be well-suited for the real-time quantification of such lipids using compact mass spectrometry systems or biosensors.

Hormonal profiling of FF further complements this personalized approach. For example, elevated concentrations of luteinizing hormone (LH) and vascular endothelial growth factor (VEGF) in FF have been linked to better fertilization outcomes and embryo development, while variations in matrix metalloproteinases (MMPs) have been associated with follicular remodeling and oocyte competence [75]. These findings suggest that an integrated, multi-marker FF analysis pipeline, facilitated by sonication-based extraction, could provide a comprehensive biochemical fingerprint of follicular health, enabling real-time optimization of IVF strategies.

By shifting biomarker evaluation from retrospective analysis to real-time diagnostics, sonication-enhanced FF profiling holds the promise of revolutionizing patient-specific infertility care. It allows clinicians to make dynamic decisions during IVF procedures, from oocyte selection to tailored stimulation protocols, thereby improving the likelihood of successful implantation and pregnancy. Table 12 summarizes the key follicular fluid biomarkers for personalized infertility treatment and the role of sonication-based analysis.

### 7.3. Bridging Research and Clinical Practice

Despite major advances in molecular profiling of follicular fluid (FF), a persistent gap remains between laboratory research and clinical infertility management. The integration of sonication-based extraction into diagnostic pipelines offers a translational bridge, allowing for the conversion of omics-level findings into actionable clinical tools. In particular, sonication facilitates consistent and high-yield extraction of proteins, metabolites, and lipids from FF, enabling the development of validated biomarker panels with robust clinical utility [76,77].

One of the key translational advantages lies in standardized biomarker quantification. By minimizing inter-sample variability, sonication supports the establishment of reproducible diagnostic thresholds for oocyte quality, embryo viability, or ovarian response [78]. Furthermore, sonication workflows are amenable to automation and scaling, making them suitable for clinical laboratories where throughput, reproducibility, and cost-effectiveness are essential [79].

The emerging paradigm of real-time, intra-procedural biomarker analysis, such as during oocyte retrieval in IVF cycles, may also be supported by portable, microfluidics-integrated sonication devices. These tools hold potential for point-of-care biomarker assessment, especially when integrated with rapid MS-based detection or biosensor platforms [80]. Early pilot studies have demonstrated the feasibility of analyzing FF within 30 min of collection, opening the possibility for real-time clinical decision-making [81].

To further close the translational gap, collaborative efforts among reproductive biologists, analytical chemists, and clinicians are needed. Multi-center validation studies using standardized sonication protocols, harmonized sample handling, and AI-assisted data interpretation will be essential to accelerate the adoption of FF-based diagnostics in ART [82]. Table 13 below summarizes the sonication-facilitated advances in clinical FF analysis.

**Figure 4:**
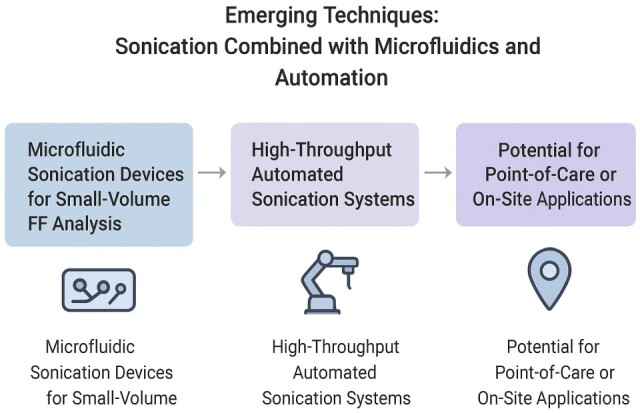
The emerging techniques related to sonication.

Figure 4 summarizes the emerging techniques related to sonication.

## 8. Challenges and Limitations of Sonication in FF Analysis

Although sonication has emerged as a powerful tool in the extraction and processing of ovarian follicular fluid (FF) for biomolecular analysis, its broader implementation, particularly in clinical settings, is constrained by several technical and procedural limitations. This section outlines these critical challenges, focusing on sample variability, biomolecular stability, reproducibility issues, and integration into assisted reproductive technologies (ART) workflows.

One of the most prominent challenges is the sample-to-sample variability that inherently affects FF analysis. FF composition is dynamic, varying with patient age, hormonal profile, follicular development stage, and overall metabolic state. As a result, even standardized sonication protocols may yield inconsistent results due to subtle differences in protein, lipid, or metabolite content, as well as physical characteristics such as viscosity or turbidity [83]. Furthermore, FF is a complex biofluid, and the lack of real-time feedback on extraction efficiency during sonication adds a layer of uncertainty when comparing outcomes across samples or studies.

A second major concern is the potential for biomolecule degradation. While controlled sonication facilitates cell lysis and protein solubilization, it also generates localized heat and can induce free radical formation, particularly under prolonged or high-power settings [84]. These physical effects can lead to oxidative degradation of lipids [85], fragmentation or denaturation of proteins [86], and even chemical modifications that alter metabolite signatures [87]. Importantly, sonication parameters must be optimized according to the targeted analyte class, as conditions suitable for robust proteins may prove too harsh for fragile metabolites or low-molecular-weight compounds, leading to selective degradation or signal loss [84,85,86,87]. The delicate balance between efficient extraction and molecular preservation remains a central tension in optimizing sonication protocols.

Reproducibility and standardization present another significant limitation. Despite increasing reports of successful sonication-based extractions in biological fluids, the field lacks unified protocols with precisely defined sonication parameters, such as amplitude, pulse duration, cycle intervals, and sample cooling methods [88]. This methodological heterogeneity limits cross-study comparisons and undermines meta-analyses aiming to validate potential biomarkers.

Moreover, integrating sonication into existing ART clinical workflows is far from trivial. IVF laboratories operate under strict time constraints and sterility requirements, particularly when handling oocytes and FF in proximity. Many sonication systems, especially probe-based devices, are not optimized for cleanroom conditions, and the risk of aerosol formation or thermal damage can pose operational challenges [89]. Automated or enclosed sonication platforms suitable for point-of-care biomarker extraction are still largely in the prototyping phase, making clinical translation difficult without further miniaturization and workflow compatibility [90]. Table 14 summarizes the key limitations in sonication-based FF analysis and their implications.

Despite these challenges, efforts to refine sonication protocols, improve instrumentation, and develop hybrid techniques combining ultrasound with microfluidics or enzyme-assisted extraction continue to show promise. However, achieving the level of control and reliability required for routine clinical deployment will depend on rigorous validation, inter-laboratory standardization, and careful consideration of patient-specific biological variability.

## 9. Future Perspectives

The use of sonication for the extraction and analysis of ovarian follicular fluid (FF) has yielded promising results in both research and preclinical domains. However, transitioning from a powerful experimental tool to a robust, clinically deployable platform demands a multidimensional roadmap. Future efforts must focus on protocol standardization, integration of multi-omics approaches, AI-enabled diagnostics, and alignment with regulatory frameworks to unlock the full diagnostic and therapeutic potential of FF analysis in reproductive medicine.

### 9.1. Standardizing Sonication Protocols for Clinical Laboratories

A major obstacle to the clinical adoption of sonication-assisted FF analysis lies in the absence of standardized operational protocols. Currently, variations in sonicator type (e.g., probe vs. bath), pulse durations, duty cycles, power output, and cooling methods contribute to substantial inter-laboratory variability [91]. Such discrepancies hinder reproducibility, regulatory approval, and the establishment of clinically actionable benchmarks.

Future studies should prioritize the development of guideline-based protocols similar to those used in clinical histopathology or hematology, with harmonized calibration routines and internal quality controls [92]. Moreover, integrating real-time temperature feedback and power modulation systems may prevent biomolecular degradation while maintaining high extraction efficiency, which is a key feature for applications involving sensitive lipid or protein targets [93].

International collaborative initiatives, potentially coordinated by professional bodies such as the International Federation of Clinical Chemistry (IFCC) or the European Society of Human Reproduction and Embryology (ESHRE), could facilitate the establishment of consensus protocols for sonication specifically adapted to reproductive biofluids. Such guidelines should explicitly account for the limited sample volumes typically associated with oocyte retrieval procedures.

### 9.2. Multi-Omics Integration: Proteome–Metabolome–Lipidome Synergy

The clinical utility of FF analysis will be greatly enhanced by transitioning from single-analyte detection to multi-omics profiling, encompassing proteomic, metabolomic, and lipidomic layers. Sonication offers the unique advantage of enabling simultaneous extraction of structurally diverse biomolecules from limited-volume samples, making it ideally suited for such integrative approaches [94].

Multi-omics datasets can reveal complex molecular networks and pathway perturbations that are not apparent when analyzing proteins or metabolites alone. For example, coupling shotgun proteomics with lipidomic fingerprinting of FF may identify key regulators of oocyte maturation or endometrial receptivity [95]. The metabolomic layer can then provide functional context to these molecular signatures.

Emerging analytical platforms, such as liquid chromatography–mass spectrometry (LC-MS/MS) and capillary electrophoresis–mass spectrometry (CE-MS), are becoming increasingly sensitive and miniaturized, thus more compatible with microfluidic sonication systems [96]. Standardized pipelines for sample preparation, data normalization, and bioinformatics analysis will be essential for translating these multi-omics datasets into clinically meaningful biomarkers.

### 9.3. Synergy with AI-Based Predictive Diagnostics

The high-dimensional data generated from multi-omics FF analysis naturally lends itself to artificial intelligence (AI) and machine learning (ML)-based predictive modeling. AI has already demonstrated superior performance in embryo selection and IVF outcome prediction compared to conventional criteria [97].

By feeding curated proteomic and metabolomic features from sonicated FF samples into ML algorithms, it may be possible to construct diagnostic classifiers that predict oocyte quality, fertilization likelihood, or even endometrial receptivity in real time. Such models could further be integrated with patient metadata, ultrasound imaging, and hormonal profiles to build comprehensive fertility dashboards [98].

The coupling of automated sonication platforms with AI-powered analysis engines [99] would enable point-of-care diagnostics, reducing dependence on centralized laboratories and shortening the clinical decision-making loop. These systems must, however, be validated against large, diverse patient cohorts to avoid algorithmic bias and ensure clinical generalizability.

### 9.4. Regulatory and Translational Considerations

Despite its technical promise, regulatory clearance remains a critical bottleneck for widespread clinical use of sonication-based FF analysis. The field currently lacks dedicated classification under major regulatory bodies such as the FDA or EMA, which complicates approval pathways for sonication devices as well as associated diagnostics [100].

To address this, manufacturers and researchers must work proactively with regulatory agencies to define quality metrics, risk assessment protocols, and performance validation standards. This includes demonstrating not only analytical sensitivity and specificity, but also clinical utility in terms of improved ART outcomes.

Furthermore, economic feasibility and scalability must be considered early in development. The design of cost-effective, disposable microfluidic cartridges for FF sonication, coupled with modular AI platforms, could offer an accessible solution even in resource-limited settings [100].

Translational research must also focus on patient acceptability and clinician usability, ensuring that workflow changes are minimal and integration into existing IVF protocols is seamless. Collaborations between academic centers, fertility clinics, industry partners, and regulatory agencies will be essential for moving beyond proof-of-concept to actual bedside implementation.

**Figure 5:**
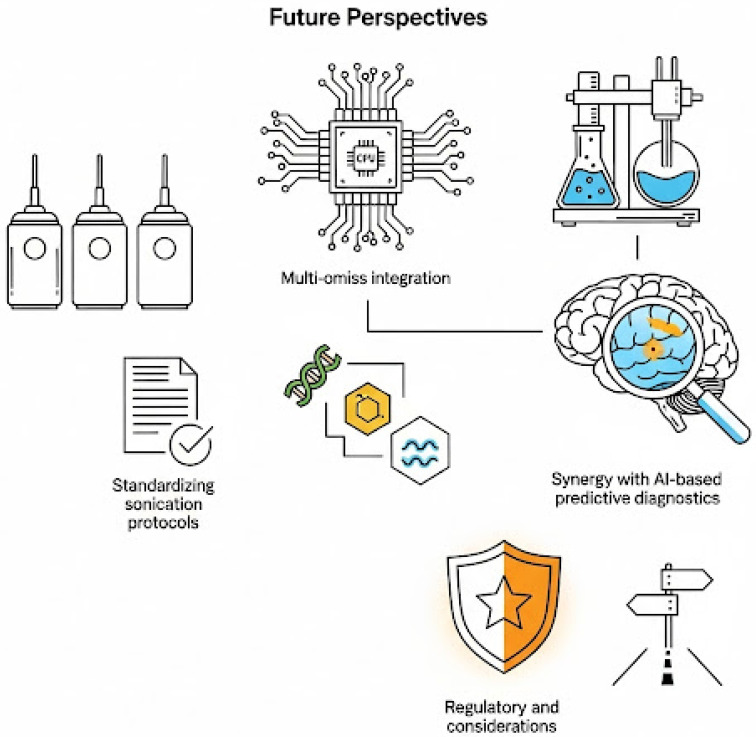
The Future Perspectives of using sonication in FF analysis.

The future perspectives of using sonication in FF analysis is depicted in Figure 5.

## 10. Conclusions

Ovarian follicular fluid represents a complex and information-rich biological matrix, harboring a wide spectrum of biomolecules, proteins, metabolites, lipids, extracellular vesicles, and hormones, that collectively reflect the intrafollicular environment and influence oocyte competence. The integration of FF analysis into infertility diagnostics and ART has the potential to enhance our understanding of ovarian physiology and improve clinical outcomes. However, the heterogeneity of FF composition and the challenges associated with its preparation have long impeded reproducible and high-resolution molecular profiling.

Sonication-based extraction has emerged as a transformative approach in this context, offering a rapid, solvent-minimizing, and highly efficient strategy for releasing biomolecules from viscous and heterogeneous FF samples. Through acoustic cavitation, sonication disrupts molecular aggregates and cell-derived vesicles, thereby improving the solubilization of proteins, release of metabolites, and recovery of lipid fractions. Optimized sonication parameters, including frequency, amplitude, duty cycle, and treatment duration, are critical to maximizing extraction efficiency while minimizing thermal or mechanical degradation of analytes [101].

Applications in proteomics, metabolomics, and lipidomics demonstrate that sonication can significantly enhance the depth and reproducibility of FF molecular profiles. These improvements are especially relevant for biomarker discovery pipelines aimed at predicting oocyte developmental competence, stratifying infertility etiologies, and guiding personalized ART interventions [102]. Moreover, the coupling of sonication with advanced mass spectrometry workflows, microfluidic platforms, and automated processing systems paves the way for high-throughput, small-volume analyses suitable for both research and clinical settings.

The accurate quantification and profiling of these complex, low-abundance components, including EVs and critical immune modulators, present a steep analytical challenge. Sonication, through its powerful mechanical energy, is uniquely capable of ensuring the efficient release of EV cargo and the thorough solubilization of associated surface proteins from the viscous FF matrix, which is essential for comprehensive multi-omic characterization [103,104]. Therefore, the successful integration of sonication protocols is paramount for achieving the required analytical depth to precisely map these biological pathways, enabling the development of the next generation of diagnostics and nanotherapeutics for conditions like POI and ovarian aging [105].

The need for this improved analytical depth is underscored by recent findings detailing the molecular complexity of reproductive disorders. For example, research into the pathophysiology of PCOS reveals critical signaling pathways mediated by EVs. Reference [103] identifies that adipocyte-derived EVs carrying miR-26b inhibit viability and promote apoptosis in cumulus cells, directly impairing ovarian function. This finding highlights that successful diagnosis and treatment rests on identifying these specific, low-abundance molecular messengers, a task made feasible by sonication’s ability to thoroughly release EV cargo from the viscous FF matrix. Furthermore, as systematic reviews confirm the complex and heterogeneous nature of PCOS [106,107], focusing on lifestyle modifications and the influence of the gut/vaginal microbiome, respectively, the demand for high-resolution, multi-omic diagnostic tools is paramount. Sonication-based analysis is uniquely positioned to deliver the necessary data resolution to accurately stratify these complex patient populations and ultimately guide true precision reproductive medicine.

Translating this analytical capability into clinical utility is already underway, with follicular fluid (FF) biomarkers being utilized to assess oocyte developmental competence, predict fertilization success, and guide personalized embryo selection strategies, leading directly to improved IVF cycle modulation [108,109,110].

Nevertheless, several challenges remain. Variability in sonication protocols across laboratories hampers reproducibility and hinders inter-study comparisons. Risks of sample overheating and biomolecule denaturation necessitate careful parameter optimization and temperature control. Furthermore, translating sonication-assisted FF analysis into routine clinical practice will require robust validation, regulatory clearance, and the development of standardized operating procedures [111].

Looking ahead, the synergy of sonication with multi-omics strategies, integrating proteomic, metabolomic, and lipidomic data, offers a more holistic representation of follicular physiology. When combined with artificial intelligence and machine learning algorithms, these datasets could underpin predictive models for IVF success, paving the way for precision reproductive medicine. The alignment of technological innovation with regulatory and translational pathways will ultimately determine the speed and extent of clinical adoption.

In conclusion, sonication-based extraction stands at the forefront of innovation in FF analysis, bridging the gap between basic research and clinical application. As protocols become standardized, and integration with multi-omics and AI-driven diagnostics advances, this technique holds the promise of redefining biomarker discovery and improving the personalization of infertility treatment strategies worldwide.

## Figures and Tables

**Table 1:** Suggested optimal sonication parameters for FF extraction.

Parameter	Suggested Range	Reference
Frequency	20–30 kHz (strong cavitation)	[40]
Amplitude (Power)	40–60%	[41]
Total Time	3–6 min (pulsed mode)	[41,42]
Pulse Duration	20–60 s with 30–60 s cooling intervals	[41,42]
Cooling Method	Ice bath or chilled rack	[43]
Buffer	8 M Urea or mild detergent (e.g., 0.1% SDS)	[42,44]
Sample Volume	100–500 µL per tube	[42]
Optimization	Western blot or peptide yield assessment	[45]

**Table 2:** Advantages of Sonication-Based Extraction.

Advantage	Description	Representative Study/Reference
Rapid homogenization	Acoustic cavitation produces high shear, dispersing FF components quickly, far faster than centrifugation or passive precipitation methods	[45]
Enhanced biomolecule release	Higher protein and metabolite yields; e.g., ultrasound-assisted extraction in algae achieved 1.32× recovery in ~10 min vs. hours in controls [46]	[46]
Minimal solvent use	Efficient extraction with smaller solvent volumes reduces costs and toxicity compared to multistep centrifugation or ultrafiltration	[46]

**Table 3:** The artifacts and mitigation strategies for sonication in FF extraction.

Artifact	Cause/Effect	Mitigation Strategy	Representative Study/Reference
Sample heating	Cavitation-induced heat → bulk temp > 30–35 °C causing protein unfolding	Pulse sonication, ice bath, use of cooling holders, temperature sensors	[35,47]
Protein denaturation/aggregation	High amplitude/time → conformational changes, β sheet amyloid-like aggregates	Optimize amplitude/duration, monitor via CD or SDSPAGE to detect aggregation	[48,49]
Poor reproducibility	Variable sonotrode positioning, sample volume, temperature control → batch-to-batch inconsistency	Standardize protocols, instrument calibration, replicate controls, temperature feedback systems	[50]

**Table 4:** The benefits of sonication in FF proteomic workflows.

Benefit	Description	Representative Example/Reference
Enhanced solubilization	Direct disruption of lipid–protein and EV matrices in FF improves protein dissolution into lysis buffer	Ultrasonication used for resuspending FF protein pellet on ice at 20% power, 50% pulse [1]
Improved digestion efficiency	Homogeneity and accessibility reduce missed cleavages, increasing peptide yield and identification rates	Consistent digestion after sonicated resuspension; enables robust LC–MS analysis [11]
Expanded proteome coverage	Extraction of low-abundance proteins and signaling molecules beyond those captured with traditional methods	Prior proteomic surveys found 742 proteins, 413 novel; sonication may further increase yield [51]

**Table 5:** The sonication benefits in FF metabolomics & lipidomics.

Benefit	Description	Representative Study/Reference
Improved metabolite recovery	Sonication disrupts EVs and lipidprotein matrices, releasing amino acids, steroids, energy metabolites	Metabolomic profiling in PCOS and endometriosis cohorts identifying proline, glycine, cortisol changes [52,53]
Expanded lipid coverage	Enhanced extraction of low-abundance lipids (LPC, PI, SM, TG, ChE) improves detection in lipidomics workflows	Lipidomics in PCOS and OHSS patients showing differential lipid species predictive of IVF outcomes [7,54].
Increased analytical sensitivity	Higher signal intensity and reduced noise across metabolites and lipids improve statistical discrimination	LC–MS models achieving AUC ~0.80 for biomarkers like prostaglandin E_2_, LPCs in PCOS FF [54]
Better reproducibility	Standardized sonication parameters (temperature, duration) reduce batch variability across small-volume FF samples	Cohort LC–MS comparisons across samples with sonication-based protocols [52]

**Table 6:** Sonication Benefits in FF Lipidomic Profiling.

Benefit	Description	Representative Study/Reference
Higher lipid recovery	Sonication disrupts EVs and protein–lipid complexes, enhancing extraction of phospholipids, sphingolipids, TGs, and LysoPCs	Endometriosis vs. control FF showing elevated PC, SM species [55]
Broader lipid class coverage	Improved detection of low-abundance lipids (LPC, CE, PI) relevant to PCOS, OHSS, unexplained infertility	Lipidomics in PCOS showing differential prostaglandin E_2_, LPCs predictive of oocyte competence [56]; age-related shifts in LysoPC, AA [56]
Enhanced analytical sensitivity	Sonication-prepped extracts yield stronger MS signals and better discrimination in multivariate models	PCOS FF model with AUC ≈ 0.80 for LPC and prostaglandin markers [56]
Improved reproducibility	Ice bath sonication ensures consistent disruption across small volume, heterogeneous FF samples early in extraction	Protocols recommending sonication for improved extraction consistency across IVF cohort samples

**Table 7:** The benefits of sonication coupled with mass spectrometry for FF analysis.

Workflow Aspect	Sonication Benefit	Outcome/Example	Reference
Proteomic LC–MS/MS	Improved protein solubilization and digestion consistency		[58]
Metabolomics & Lipidomics via UHPLC–MS/MS	Efficient release of both polar metabolites and diverse lipid species	Detection of PC, LPC, SM, TG, ChE shifts in PCOS/OHSS FF	[59,60]
High-throughput proteomics (DIA/TMT)	Uniform extraction across plates via automated sonication reduces batch variability	Cohort-scale quantification with CV < 5%	[62]
Shotgun lipidomics by direct infusion	Sonication-prepared extracts minimize matrix effects for rapid lipid profiling	Hundreds of lipid species profiled per sample	[62]

**Table 8:** Key developments in microfluidic sonication platforms.

Application	Principle and Sonication Role	Outcome/Relevance to FF Analysis
Cell-free DNA extraction	Acoustofluidic bubble-based mixing enhances analyte release in microliters	~10× increase in DNA recovery from <500 μL plasma [63]
Liposomal nanoparticle synthesis	Sonication in microchannels refines particle size and uniformity	Demonstrates acoustic control over nanoscale extraction
MicroChIP/MeDIP assays	Acoustic streaming improves mixing and DNA shearing in tiny reaction chambers	High sensitivity, <1 h workflows from minimal input [65]

**Table 9:** High throughput automated sonication for FF analysis.

Platform	Key Features	Relevance to FF Workflows	References
Hielscher UIP400MTP	Non-contact focused ultrasonication in standard multi-well plates; uniform cavitation; programmable parameters	Enables reproducible, scalable FF extraction in plate-based workflows	[66]
Covaris LE220-plus/LE220R/R230	High-throughput AFA^®^ sonication with robotic compatibility (1–96 samples)	Supports integration into liquid-handling systems for automated FF prep	[67]
Covaris Sonication STAR	Fully automated sonication within integrated robotic and extraction platform	Demonstrates feasibility of walk-away multimodal extraction from precious clinical samples	[68]

**Table 10:** The POC microfluidic devices with sonication for FF analysis.

Feature	Description	Relevance to FF Analysis
Integrated microfluidic POC	Complete “sample-in, answer-out” lab-on-chip systems with enclosed workflows	Enables compact, sterile analysis directly at point-of-care
Rapid processing & low volume	Handling of small sample volumes with swift turnaround times	Preserves precious FF, supports real-time clinical decisions
Smartphone or on-site readout	Optical/electronic detection via mobile interfaces	Real-time results capture and communication in clinic

**Table 11:** Sonication-driven enhancements in biomarker discovery.

Enhancement	Impact on Biomarker Discovery	Supporting Example/Reference
Heightened analytical sensitivity	Enhanced release and detection of low-abundance lipids and metabolites	Differential lipid signatures in infertile vs. fertile FF profiles [7]
High reproducibility	Consistent biomarker quantification across large sample sets	LPC validated across cohorts using ELISA [71]
Reliable metabolite differentiation	Enables detection of disease-specific metabolic shifts in FF	Altered glucose, lactate, pyruvate in endometriosis or DOR [72]

**Table 12:** The key follicular fluid biomarkers for personalized infertility treatment and the role of sonication-based analysis.

Biomarker	Associated Outcome	Diagnostic Performance	Sonication Advantage	Reference
Leptin	Oocyte maturity	Threshold: 16 ng/mL Sensitivity: 70% Specificity: 91% AUC: 0.829	Enhances solubilization of hormone-bound proteins	[73]
Resolvin E1 (RvE1)	Oocyte quality	Threshold: 8.96 pg/mL Specificity: 97%, AUC: 0.75	Efficient lipid release for metabolomic screening	[74]
LH & VEGF	Fertilization and embryo development success	Elevated levels linked to improved outcomes	Preserves bioactivity of proteins during release	[75]
MMPs	Follicular remodeling and oocyte competence	Correlated with maturation and implantation potential	Enables multiplexed protein profiling from small volumes	[75]

**Table 13:** Sonication-facilitated advances in clinical FF analysis.

Translational Challenge	Sonication-Enabled Solution	Clinical Implication	Reference
Inconsistent biomarker extraction	Enhanced reproducibility and yield across FF samples	Reliable patient stratification and IVF outcome prediction	[76,77]
Lack of standardized protocols	Tunable and automatable workflows	Suitable for high-throughput clinical labs	[78,79]
Delayed laboratory results	Potential for real-time biomarker analysis via microfluidics	Immediate intra-cycle decision-making	[80,81]
Fragmented interdisciplinary collaboration	Facilitate standardization across research and clinical teams	Accelerated biomarker validation and deployment	[82]

**Table 14:** The key limitations in sonication-based FF analysis and their implications.

Challenge	Cause/Mechanism	Implications
Sample-to-sample variability	Variations in FF composition and fluid properties	Inconsistent extraction outcomes across individuals
Biomolecular degradation	Heat, shear stress, and radical formation during sonication	Risk of lipid oxidation, protein denaturation, and metabolite loss
Reproducibility and standardization	Lack of consensus on sonication protocols	Difficulties in cross-study validation and clinical translation
Clinical workflow integration	Equipment bulk, heat generation, and sterility concerns	Limited feasibility in IVF labs and ART settings

## Data Availability

No data was generated for this article.
